# Quality of life and intrinsic capacity in patients with post-acute COVID-19 syndrome is in relation to frailty and resilience phenotypes

**DOI:** 10.1038/s41598-023-29408-z

**Published:** 2023-06-02

**Authors:** Giovanni Guaraldi, Jovana Milic, Sara Barbieri, Tommaso Marchiò, Agnese Caselgrandi, Federico Motta, Bianca Beghè, Alessia Verduri, Michela Belli, Licia Gozzi, Vittorio Iadisernia, Matteo Faltoni, Giulia Burastero, Andrea Dessilani, Martina Del Monte, Giovanni Dolci, Erica Bacca, Giacomo Franceschi, Dina Yaacoub, Sara Volpi, Alice Mazzochi, Enrico Clini, Cristina Mussini

**Affiliations:** 1 Department of Surgical, Medical, Dental and Morphological Sciences, University of Modena and Reggio Emilia, Largo del Pozzo, 71, 41124 Modena, Italy; 2 Department of Infectious Diseases, Azienda Ospedaliero-Universitaria, Policlinico of Modena, Modena, Italy; 3 University of Modena and Reggio Emilia, Modena, Italy; 4 Respiratory Unit, Azienda Ospedaliero-Universitaria, Policlinico of Modena, Modena, Italy; 5 Department of Medical and Surgical Sciences for Children and Adults, University of Modena and Reggio Emilia, Modena, Italy

**Keywords:** Viral infection, Geriatrics

## Abstract

The objective of this study was to characterize frailty and resilience in people evaluated for Post-Acute COVID-19 Syndrome (PACS), in relation to quality of life (QoL) and Intrinsic Capacity (IC). This cross-sectional, observational, study included consecutive people previously hospitalized for severe COVID-19 pneumonia attending Modena (Italy) PACS Clinic from July 2020 to April 2021. Four frailty-resilience phenotypes were built: “fit/resilient”, “fit/non-resilient”, “frail/resilient” and “frail/non-resilient”. Frailty and resilience were defined according to frailty phenotype and Connor Davidson resilience scale (CD-RISC-25) respectively. Study outcomes were: QoL assessed by means of Symptoms Short form health survey (SF-36) and health-related quality of life (EQ-5D-5L) and IC by means of a dedicated questionnaire. Their predictors including frailty-resilience phenotypes were explored in logistic regressions. 232 patients were evaluated, median age was 58.0 years. PACS was diagnosed in 173 (74.6%) patients. Scarce resilience was documented in 114 (49.1%) and frailty in 72 (31.0%) individuals. Predictors for SF-36 score < 61.60 were the phenotypes “frail/non-resilient” (OR = 4.69, CI 2.08–10.55), “fit/non-resilient” (OR = 2.79, CI 1.00–7.73). Predictors for EQ-5D-5L < 89.7% were the phenotypes “frail/non-resilient” (OR = 5.93, CI 2.64–13.33) and “frail/resilient” (OR = 5.66, CI 1.93–16.54). Predictors of impaired IC (below the mean score value) were “frail/non-resilient” (OR = 7.39, CI 3.20–17.07), and “fit/non-resilient” (OR = 4.34, CI 2.16–8.71) phenotypes. Resilience and frailty phenotypes may have a different impact on wellness and QoL and may be evaluated in people with PACS to identify vulnerable individuals that require suitable interventions.

## Introduction

COVID-19 is a complex disease with long-term sequelae after the resolution of acute-phase symptoms^1^. Several cluster of symptoms, characterized by significant deterioration of quality of life and increased risk of death after the resolution of infectious symptoms, have been grouped under the umbrella term of post-acute COVID syndrome (PACS)^2^. The prevalence of PACS vary from 10% in the people with previously documented infection^3^ up to 50–80% among people who were hospitalized due to severe COVID-19 pneumonia^4–9^. A large observational cohort study described that 1 in 3 patients had one or more features of PACS after 3 to 6 months of an acute infection. The most common symptoms included: abnormal breathing (18.7% during the first semester after the infection), fatigue/malaise (12.8%), chest/throat pain (12.6%), headache (8.7%), other pain (11.6%), abdominal symptoms (15.5%), myalgia (3.2%), cognitive symptoms (7.8%), and anxiety/depression (22.8%)^10^.

Understanding the impact of COVID-19 on progression of comorbid chronic disease, aging, and quality of life remains one of the most important issues in PACS characterization. This syndrome, similarly, to other chronic inflammatory condition, can be described as an accentuated aging process and as such can depict the damage and repair mechanism balance as well as, at a patient centered approach level, in terms of health related-quality of life (HRQoL) and well-being. Damage and repair mechanisms belong to the constructs of frailty and resilience, while well-being to the multidimensional conceptualization of HRQoL and intrinsic capacity (IC).

Frailty is a state of an increased vulnerability that negatively impacts aging trajectories and it may be depicted by reduced strength and decline of physiological compensation^11^.This state results in increased risk of unfavorable health outcomes^11,12^. In the COVID-19 setting, several studies found correlation between frailty and severity of the disease during the acute phase^13^. Screening for frailty in patients with COVID-19 allows to better predict mortality or adverse outcomes that may occur after hospital discharge that be depicted by PACS^14,15^. Although the relationship between frailty and PACS remains largely unexplored, it is very indicative that acute COVID-19 as a stressor may induce or worsen frailty. In order to capture the impact of the stressor, the construct of resilience may be very helpful.

Resilience represents the capacity to fully or partially recover after exposure to stressor, such as COVID-19. It is a dynamic trajectory over time in which post-stress equilibrium may be the same as the initial one or different^16^. While frailty describes accumulation of deficits, the resilience represents the capacity to reach a new equilibrium after exposure to stress due to COVID-19. Thus, frailty and resilience may be complementary concepts that capture different domains of patients’ vulnerability during acute COVID-19 infection and PACS^17^.

In a clinical scenario in which the treatment of PACS is scarce, the implementation of a patient centered approach through the description of HRQoL and IC domains will help to address patient needs and provide additional information regarding everyday functioning.

Health Related Quality of life (HRQoL) is a multi-dimensional concept that includes domains related to physical, mental, emotional, and social functioning. A related concept of HRQoL is well-being, which assesses the positive aspects of a person’s life, such as Intrinsic capacity^18^. Assessing intrinsic capacity is both a multidisciplinary and a multidimensional process, designed to evaluate the individual’s biology on the basis of five functional domains: locomotion, cognition, psychology, vitality, and sensory^19^. Description of these domains is of a paramount importance, as it may help to optimize patient-centered approaches in the management of people with PACS^20^.

The objective of this study was to characterize frailty and resilience phenotypes in people with previous severe COVID, evaluated for PACS, which may differently impact health-related quality of life (HRQoL) and intrinsic capacity (IC).

## Methods

### Study design

This was a cross-sectional, observational study that included consecutive patients attending Modena PACS Clinic (MPC) from 15 July 2020 to 30 April 2021. MPC is a multidisciplinary referral center established after the first wave of COVID-19 pandemic in Italy in which patients are screened for signs and symptoms of PACS. Data were obtained from electronic health records and complied fully with Italian law on personal data protection and the ethics committee of the *Area Vasta Nord Emilia Romagna* who approved the study (396/2020/OSS/AOUMO-Cov-2 MO-Study).

Inclusion criteria for the present study were age ≥ 18 years, previous hospitalization for severe COVID-19 pneumonia, willingness and capacity to complete electronic questionnaires via web or with i-pad. All questionnaires were validated and administered in Italian. Patients were evaluated at least three months after hospital discharge.

Demographic, anthropometric, hospitalization and PACS signs and symptoms were collected at the same day of the visit at MPC.

PACS diagnosis was considered after a minimum of 12 weeks after the onset of SARS-CoV-2 infection^21^ when, at least one of the following cluster symptoms were present: neurocognitive (brain fog, dizziness, loss of attention, confusion), autonomic (chest pain, tachycardia, palpitations), gastrointestinal (diarrhea, abdominal pain, vomiting), respiratory (general fatigue, dyspnea, cough, throat pain), musculoskeletal (myalgias, arthralgias), psychological (post- traumatic stress disorder, anxiety, depression, insomnia), metabolic (Non-alcoholic fatty liver disease-NAFLD assessed with transient elastography using a CAP cutoff > 248 dB/m), sensory (ageusia, anosmia, hearing loss) and dermatological (hair loss, skin rashes).

### Covariates

*Resilience* was assessed using the Connor Davidson resilience scale (CD RISC-25). The questionnaire covers the following issues: personal competence, standards and tenacity, trust in its instincts, tolerance of negative effect, acceptance of change, feeling of control and spiritual influences. The responses were evaluated on a five-point Likert scale ranging from 0 to 4: not true at all (0), rarely true (1), sometimes true (2), often true (3), and true nearly all of the time (4). These ratings result in a number between 0 and 100. For the purpose of our study, resilience was defined as CD-RISC-25 score > 60, above the median of the study population^22^.

*Frailty Phenotype (FP)* was assessed using Fried frailty criteria. The tool leads to frailty diagnosis when at least 3 of the 5 items are present, including unintentional weight loss (self-reported), exhaustion (self-reported), low energy expenditure, walking speed, weak grip strength. In this study, people with FP scores 0, 1 and 2 were considered fit, while those with FP > 2 frail.

According to our pre-plan analyses four frailty-resilience phenotypes were built: “fit/resilient”, “fit/non-resilient”, “frail/resilient” and “frail/non-resilient”, based on previously reported cut-offs for both scores.

### Outcomes

*Health-related quality of life (HRQoL)*, assessed by Short Form 36 (SF-36) Health Survey Questionnaire and by EQ-5D-5L questionnaire. Short Form 36 (SF-36 Health Survey Questionnaire) Health Survey Questionnaire is a 36-item scale, which measures nine domains of health status: physical functioning (ten items); physical role limitations (four items); bodily pain (two items); general health perceptions (five items); energy/vitality (four items); social functioning (two items); emotional role limitations (three items), mental health (five items) and health change (one item). A scoring algorithm is used to convert the raw scores into the nine dimensions listed above. The scores are transformed to range from zero (in which the respondent had the worst health) to 100 (in which the respondent had the best health). For each domain, an outcome measure was defined as the score below and above the average, that was previously standardized^23,24^. The original interpretation describes separately all nine domains. To estimate overall quality of life using SF-36, we considered the contribution of each domain to total mean score, e.g. physical functioning comprises 10 out of 36 items, that equals to 27.8%, emotional well-being and general health comprise 5 each out of 36 items that equals to 13.9%, role limitations due to physical health and energy/fatigue comprise 4 each out of 36 items that equals to 11.1%, role limitations due to emotional problems comprise 3 out of 36 items that equals to 8.33%, social functioning and pain comprise 2 each out of 36 items that equals to 5.56% and finally, health change comprises only 1 out of 36 item that equals to 2.78%. Using the means for each domain and previously described percentages, a total mean score for quality of life was estimated^23^ based on the following calculation:

$$\begin{aligned} {\text{Standard}}\;{\text{total}}\;{\text{mean}}\;{\text{score}} = & 0.{278}{ \times }{7}0.{61 + }0.{139}{ \times }{65}.{78 + 39}{ \times }{56}.{99} \\ & \quad { + }0.{111}{ \times }{52}.{97 + }0.{111}{ \times }{52}.{15 + }0.0{83}{ \times }{7}0.{38} \\ & \quad { + }0.0{56}{ \times }{78}.{77 + }0.0{56}{ \times }{7}0.{77 + }0.0{{28}} {\times } {{59}}.{14} = {61}.{6}0 \\ \end{aligned}$$

Total mean scores in people with PACS were calculated using the same formula. Quality of life above the mean was defined as score of SF-36 > 61.60.

*EQ-5D-5L* evaluated the following domains: Mobility, Self-care, Anxiety and depression, Pain and discomfort, Usual activity. Each question has 5 possible answers: no problems, slight problems, moderate problems, severe problems, and extreme problems. The EQ-VAS recorded the respondent’s self-rated health from 0 to 100 on a 20 cm visual analogue scale with endpoints labelled ‘the best health you can imagine’ and ‘the worst health you can imagine’. The optimal quality of life was defined as score of EQ-5D-5L > 89.7%, as described in Spanish general population and according to EQ-5D Guide^25,26^. Spain was chosen as a country with similar socio-economic characteristics as Italy.

*Intrinsic capacity (IC)* was assessed using a 37-item IC questionnaire, developed according to WHO ICOPE guidelines^27^. This questionnaire was developed using previously validated questions relevant to the five domains of IC (Supplementary table 1). The answers to all questions were categorized as “0” or “1”, in which 0 was assigned if the answer had “negative” and 1 if the answer had “positive” connotation. The final score was calculated using the following formula: (number of “positive” items/33)*100, in which the higher score implies better IC.

### Statistical analysis

Data were expressed as mean ± standard deviation (SD) for normally distributed continuous variables, as median and interquartile range (IQR) for non-normally distributed continuous variables, and as frequencies and percentages for categorical variables. Student’s t-test and ANOVA were performed to identify statistical difference for the normally distributed continuous variables, while Mann–Whitney and Kruskal–Wallis tests were used for not normally distributed continuous variables. The χ2 (chi-squared) test was applied for categorical variables. Characteristics of people with PACS were described according to resilience and frailty separately and according to resilience-frailty phenotypes.

We developed a heatmap for categorical variables in which each line represents a single individual, and a color code was used to identify the presence or absence of a cluster, according to the frail/resilience phenotypes.

Quality of life (measured both with SF-36 and EQ-5D-5L) and intrinsic capacity, were described according to frailty-resilience phenotypes using radar graphics. All means of single questionnaires were normalized as a score from 0 to 100, in which higher score implied higher IC and QoL. Lower scores are near the center of the radar graph, while higher scores are near the periphery of the circle.

Multivariable logistic regressions were also built to investigate predictors of quality of life and Intrinsic capacity with particular attention on frailty-resilience phenotypes.

Multivariate regression models included covariates with a *P*-value < 0.05 in univariable analysis or covariates that were determined a priori to be clinically important, based on previous literature.

The statistical analysis was performed in Phyton. This study was approved by the University of Modena and Reggio Emilia ethics committee. This was a retrospective study conducted using clinical data anonymized in accordance with the requirements of the Italian Personal Data Protection Act. Patients' informed consent was deemed unnecessary by the Regional Ethics Committee of Emilia Romagna according to Italy's Legislative Decree No. 211/2003. The study was conducted according to the guidelines of the Declaration of Helsinki^28^.

### Data availability

The datasets used and/or analyzed during the current study available from the corresponding author on reasonable request.

## Results

**Figure 1:**
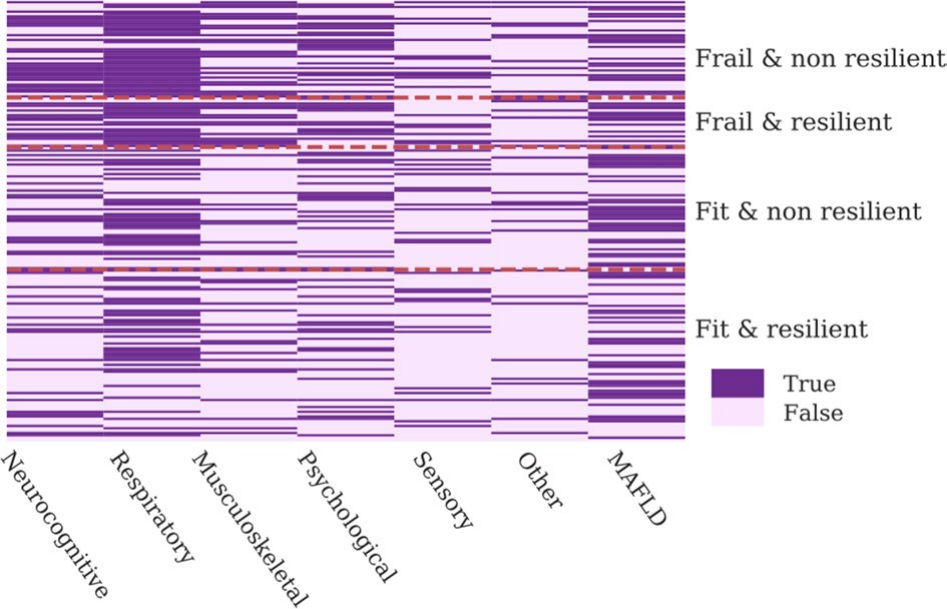
Prevalence of PACS clusters according to frail/resilience phenotypes is shown in the heatmap.

In the period July 2020–April 2021, 232 patients were evaluated at MPC, median age was 58.0 (Q1,Q3: 50.0–67.0) years. Prevalence of non-resilience was 114 (49.1%), while prevalence of frailty was 72 (31.0%). Prevalence of PACS was 173 (74.6%), specifically respiratory cluster was represented in 128 (55.2%), NAFLD 93 (40.1%), musculoskeletal 67 (28.9%), neurocognitive in 82 (35.3%), psychological 79 (34.1%), sensory 49 (21.1%), other 42 (18.1%) (Table 1 and Fig. 1).

**Table 1:** Demographic, anthropometric and clinical characteristics, comorbidities and patient-reported outcomes according to four frailty-resilience phenotypes.

	Fit & resilient N = 95 (41%)	Fit & non resilient N = 65 (28.0%)	Frail & resilient N = 23 (9.9%)	Frail & non resilient N = 49 (21.1%)	Total 232 (100%)	*P*
Demographic, anthropometric and clinical characteristics at MPC visit						
Age, years, median (Q1-Q3) [N_0_]	60.0 (51.0–66.5) [95]	58.0 (49.0–66.0) [65]	54.0 (51.5–67.0) [23]	58.0 (53.0–68.0) [49]	58.0 (50.0–67.0) [232]	0.8
Male sex, N (%)	66 (69.5%)	39 (60.0%)	11 (47.8%)	25 (51.0%)	141 (60.8%)	0.09
Body mass index, kg/m^2^, median (IQR) [N_0_]	29.1 (25.9–32.0) [79]	28.0 (25.8–31.0) [59]	30.43 (27.1–34.6) [23]	30.7 (25.5–33.9) [43]	29.3 (25.8–32.4) [204]	0.14
ASCVD risk score, median (Q1-Q3) [N_0_]	10.4 (5.3–20.4) [56]	7.7 (4.0–17.5) [39]	5.7 (4.3–10.9) [13]	8.4 (2.5–11.6) [28]	9.1 (4.4–17.6) [136]	0.41
Physical activity, N (%)						< 0.001
Low physical activity	52 (54.7%)	37 (56.9%)	21 (91.3%)	46 (93.9%)	156 (67.2%)	
Moderate physical activity	39 (41.1%)	26 (40.0%)	2 (8.7%)	3 (6.1%)	70 (30.2%)	
Intense physical activity	4 (4.2%)	2 (3.1%)	0 (0.0%)	0 (0.0%)	6 (2.6%)	
Metabolic syndrome, N (%)	11 (11.6%)	13 (20.0%)	6 (26.1%)	10 (20.4%)	40 (17.2%)	0.43
Diabetes, N (%)	10 (10.5%)	7 (10.8%)	4 (17.4%)	5 (10.2%)	26 (11.2%)	0.89
Obesity, N (%)	29 (30.3%)	19 (29.2%)	12 (52.2%)	24 (49.0%)	84 (36.2%)	0.06
PACS clusters						
Respiratory cluster, N (%)	36 (37.9%)	35 (53.9%)	18 (78.3%)	39 (79.6%)	128 (55.2%)	< 0.001
Neurocognitive cluster, N (%)	19 (20.0%)	24 (36.9%)	12 (52.2%)	27 (55.1%)	82 (35.3%)	< 0.001
Musculoskeletal cluster, N (%)	18 (19.0%)	14 (21.5%)	12 (52.2%)	23 (46.9%)	67 (28.9%)	< 0.001
Psychological cluster, N (%)	22 (23.2%)	19 (29.2%)	11 (47.8%)	27 (55.1%)	79 (34.1%)	< 0.001
Sensory cluster, N (%)	14 (14.7%)	15 (23.1%)	6 (26.1%)	14 (28.6%)	49 (21.1%)	0.22
Dermatologic cluster, N (%)	10 (10.5%)	13 (20.0%)	6 (26.1%)	13 (26.5%)	42 (18.1%)	0.07
NAFLD cluster, N (%)	31 (32.6%)	29 (44.6%)	12 (52.2%)	21 (42.9%)	93 (40.1%)	0.42
PACS syndrome, N (%)	59 (62.1%)	48 (73.9%)	22 (95.7%)	44 (89.8%)	173 (74.6%)	< 0.001
Geriatric syndromes						
Falls in the last year, N (%)	11 (11.6%)	3 (4.6%)	8 (34.8%)	13 (26.5%)	35 (15.1%)	< 0.001
Polypharmacy, N (%)	12 (12.6%)	16 (24.6%)	12 (52.2%)	17 (34.7%)	57 (24.6%)	< 0.001
Walked less in the last year, N (%)	36 (37.9%)	27 (41.5%)	18 (78.3%)	35 (71.4%)	116 (50.0%)	< 0.001
Loneliness, N (%)	9 (9.5%)	10 (15.4%)	5 (21.7%)	20 (40.8%)	44 (19.0%)	< 0.001

Supplementary table 2 details demographic, anthropometric and clinical variables in people with a without frailty. Patients with frailty had higher BMI (30.6 kg/m^2^ vs. 28.9 kg/m^2^, *P* = 0.03), lower levels of moderate physical activity (6.9% vs. 44.4%, *P* < 0.001) and higher prevalence of PACS (91.7% vs. 66.9%, *P* < 0.001). Regarding geriatric syndromes, higher burden of polypharmacy (40.3% vs. 17.5%, *P* < 0.001), loneliness (34.7 vs. 11.9%, *P* < 0.001) and falls (29.2% vs. 8.8%, *P* < 0.001) was observed (Supplementary table 2).

Prevalence of impaired resilience using CD-RISC-25 cut-off < 60 was 49.1%. Supplementary table 3 details demographic, anthropometric and clinical characteristics in people with and without resilience. Patients with impaired resilience had similar age (58.0 vs. 59.5, *P* = 0.44) and BMI (29.3 kg/m^2^ vs. 29.3 kg/m^2^, *P* = 0.88) in comparison to resilient patients. Regarding geriatric syndromes, similar burden of polypharmacy (29.0% vs. 20.3%, *P* = 0.17) and falls (14.0% vs. 16.1%) was observed, while there was significant difference in loneliness (26.3% vs. 11.9%, *P* = 0.008) (Supplementary table 3).

Table 1 shows four frailty-resilience phenotypes. Groups included 95 (41.0%) “fit and resilient”, 65 (28.0%) “fit and non-resilient”, 23 (9.9%) “frail and resilient” and 49 (21.1%) “frail and non-resilient”. No difference was found in age and sex and cardiometabolic comorbidities across the 4 phenotypes. Prevalence of obesity was higher in frail groups as well as of PACS clusters with the exemption of sensory, NAFLD and dermatologic cluster. Altogether, PACS was significantly higher in frail when compared to fit groups, respectively 91.7% vs. 64.8% (*P* < 0.001). Geriatric syndromes also, with particular regards to falls, walking less in the last year, polypharmacy and loneliness were more prevalent in the frail patients (Table 1, Supplementary table 2).

Prevalence of PACS clusters according to frail/resilience phenotypes is shown in the heatmap (Fig. 1). In detail this figure shows the distribution of each PACS cluster in a single patient, represented by a single line.

**Figure 2:**
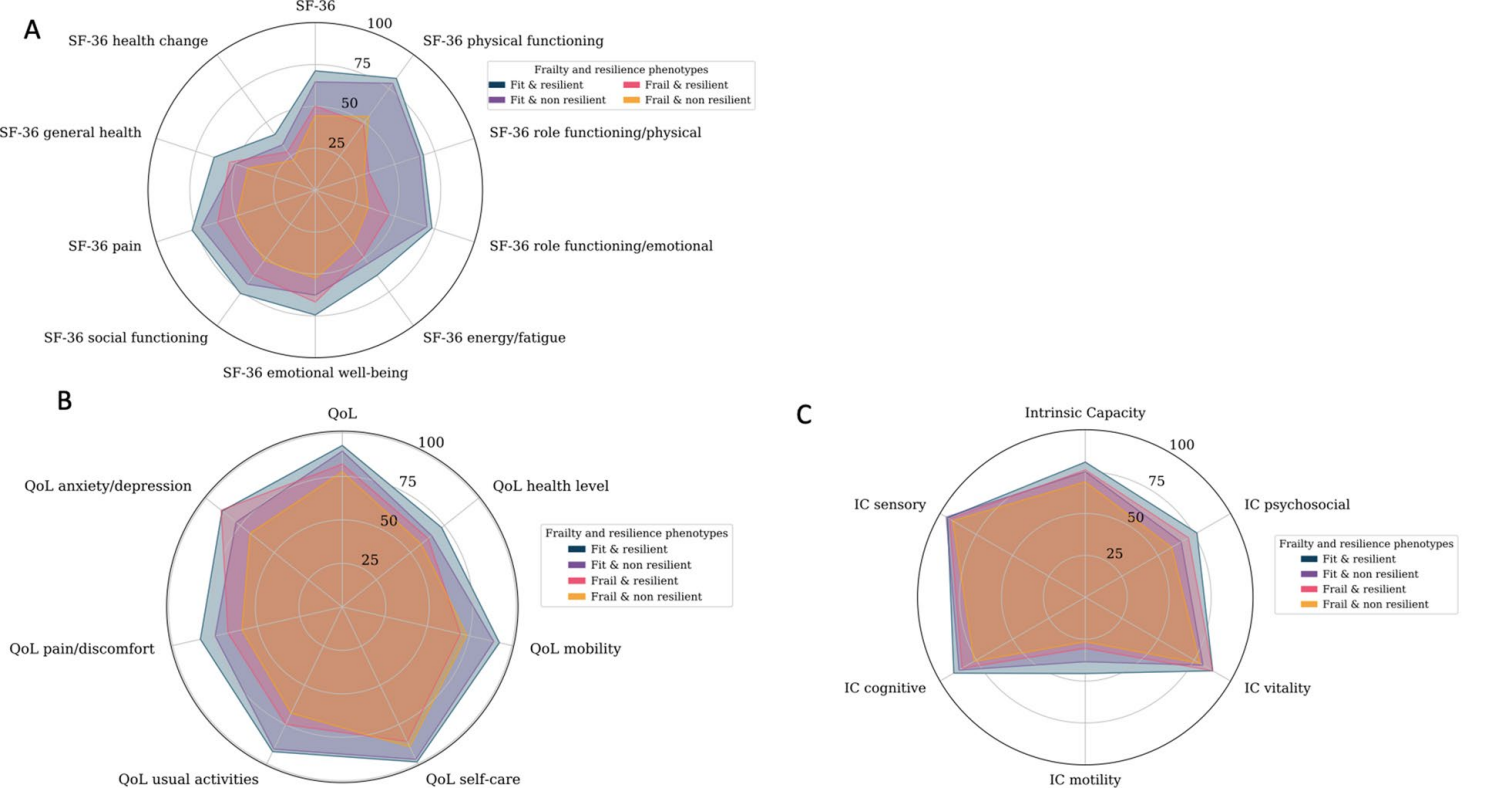
Description of SF-36 (panel A), EQ-5D-5L (panel B) and IC domain (panel C) across the phenotypes. All means single questionnaires were normalized as a score from 0 to 100, in which higher score implies better IC and QoL. Lower scores are depicted near the center of the radar graph, while higher scores are near the periphery of the circle.

With regards to study outcomes, Fig. 2 depicts in radar graphs, mean scores of each domain of EQ-5D5L, SF-36 and IC. Figures shows polygon areas for each frailty/resilience phenotypes. Progressive increase of mean scores of each domain are plotted in the vertices of polygons, from the lowest (near the center) in frail and non-resilient, to highest (towards periphery) in fit and resilient. Details of mean values or each domain are shown in Table 2. Mean values were statistically different in the majority of comparisions.

**Table 2:** Outcomes.

	Fit & resilient N = 95 (41%)	Fit & non resilient N = 65 (28.0%)	Frail & resilient N = 23 (9.9%)	Frail & non resilient N = 49 (21.1%)	Total 232 (100%)	*P*
SF-36						
Overall SF-36 score, mean (± SD) [N_0_]	71 (± 17) [95]	65 (± 16) [65]	50 (± 20) [23]	44 (± 16) [49]	62 (± 20) [232]	< 0.001
Health change, mean (± SD) [N_0_]	41 (± 23) [95]	33 (± 22) [65]	28 (± 29) [23]	22 (± 20) [49]	34 (± 24) [232]	< 0.001
Energy/fatigue, median (Q1,Q3) [N_0_]	65 (48–80) [95]	55 (40–65) [65]	50 (35–62) [23]	40 (30–50) [49]	55 (40–70) [232]	< 0.001
General health, mean (± SD) [N_0_]	64 (± 20) [95]	50 (± 18) [65]	54 (± 16) [23]	42 (± 19) [49]	54 (± 20) [232]	< 0.001
Pain, mean (± SD) [N_0_]	77 (± 22) [95]	72 (± 24) [65]	61 (± 27) [23]	49 (± 26) [49]	68 (± 26) [232]	< 0.001
Physical functioning, mean (± SD) [N_0_]	82 (± 18) [95]	79 (± 19) [65]	49 (± 26) [23]	54 (± 24) [49]	72 (± 24) [232]	< 0.001
Role limitations due to physical health, mean (± SD) [N_0_]	68 (± 39) [95]	66 (± 39) [65]	34 (± 40) [23]	31 (± 38) [49]	56 (± 42) [232]	< 0.001
Role limitations due to emotional problems, mean (± SD) [N_0_]	73 (± 34) [95]	70 (± 36) [65]	46 (± 41) [23]	33 (± 37) [49]	61 (± 39) [232]	< 0.001
Social functioning, mean (± SD) [N_0_]	76 (± 23) [95]	69 (± 21) [65]	62 (± 28) [23]	52 (± 20) [49]	68 (± 24) [232]	< 0.001
Emotional well-being, (Q1,Q3) [N_0_]	76 (64–88) [95]	64 (52–76) [65]	64 (56–78) [23]	52 (44–64) [49]	64 (55–80) [232]	< 0.001
EQ-5D-5L						
EQ-5D-5L, mean (± SD) [N_0_]	88 (± 11) [95]	83 (± 14) [65]	70 (± 23) [23]	63 (± 26) [49]	80 (± 20) [232]	< 0.001
EQ-5D-5L health score, mean (± SD) [N_0_]	73 (± 15) [95]	66 (± 16) [65]	63 (± 16) [23]	58 (± 14) [49]	67 (± 16) [232]	< 0.001
Intrinsic capacity						
Overall IC score, median (Q1,Q3) [N_0_]	79 (76–86) [95]	76 (69–83) [65]	76 (69–83) [23]	69 (66–76) [49]	76 (69–83) [232]	< 0.001
Cognition, mean (± SD) [N_0_]	91 (± 27) [95]	87 (± 28) [65]	85 (± 32) [23]	77 (± 34) [49]	86 (± 30) [232]	0.06
Mobility, mean (± SD) [N_0_]	46 (± 27) [95]	38 (± 30) [65]	30 (± 29) [23]	27 (± 24) [49]	38 (± 28) [232]	< 0.001
Psychosocial, mean (± SD) [N_0_]	77 (± 14) [95]	66 (± 17) [65]	71 (± 14) [23]	59 (± 14) [49]	70 (± 16) [232]	< 0.001
Sensory, mean (± SD) [N_0_]	95 (± 10) [95]	96 (± 8) [65]	93 (± 11) [23]	91 (± 11) [49]	94 (± 10) [232]	0.11
Vitality, mean (± SD) [N_0_]	88 (± 15) [95]	81 (± 18) [65]	88 (± 13) [23]	79 (± 16) [49]	84 (± 16) [232]	0.003

Multivariate logistic analyses were used to identify predictors of the total scores of EQ-5D5L, SF-36 and IC.

**Figure 3:**
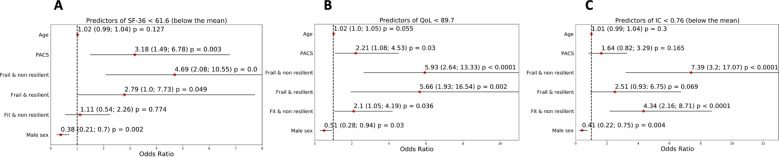
Describes predictors of health-related quality of life and intrinsic capacity. (**A**): Predictors for SF-36 Health Survey Questionnaire score <61.60. (**B**): Predictors for EQ-5D-5L <89.7%. (**C**): Predictors of impaired IC.

Predictors for SF-36 Health Survey Questionnaire score < 61.60 were the phenotypes “frail/non-resilient” (OR = 4.69, 95% CI, 2.08; 10.55, *P* < 0.01) “fit/non-resilient” (OR = 2.79, 95% CI, 1.00; 7.73, *P* = 0.049) (Fig. 3A). Predictors for EQ-5D-5L < 89.7% were the phenotypes “frail/non-resilient” (OR = 5.93, 95% CI, 2.64; 13.33, *P* < 0.001), “frail/resilient” (OR = 5.66, 95% CI, 1.93; 16.54, *P* = 0.002) (Fig. 3B). Predictors of impaired IC were “frail/non-resilient” (OR = 7.39, 95% CI, 3.20; 17.07, *P* < 0.001), and “fit/non-resilient” (OR = 4.34, 95% CI, 2.16; 8.71, *P* < 0.001) phenotypes. Male sex was negatively associated with impaired IC (OR = 0.41, 95% CI, 0.22; 0.75, *P* = 0.004) (Fig. 3C).

## Discussion

While frailty has been extensively used to describe vulnerability to adverse outcomes and mortality in patients with acute COVID-19^13,29,30^, this study focused on its characterization in the post-acute phase of the disease, captured by PACS. Frailty is here depicted jointly with resilience through frailty/resilience phenotypes, providing a new insight into the vulnerability of people hospitalized for severe COVID 19.

The 4 frailty-resilience phenotypes were differently associated with PACS and geriatric variables and more importantly had different impact on HRQoL and IC. In detail, resilience and frailty acted as complementary forces that contributed diversly to multiple domains of QoL and IC.

Characterization of phenotypes highlight a patient centered model which characterize MPC. This diagnostic and therapeutic pathway is not simply based on a multidisciplinary intervention where multiple health care providers take care of diseases and organ impairment but it also adds a multidimensional evaluation where patients reported outcomes are evaluated and discussed with the patients and functional impairments are treated in order to maximize HRQoL and intrinsic capacity.

Frailty was evaluated with “frailty phenotype” tool using a dichotomous stratification in which fit and prefrail were group together in the category “non frail” in consideration of the limited number of fit individuals. Acute SARS-CoV-2 infection and PACS may induce or worsen frailty, even in younger individuals, indicating that frailty instead of chronological age should be used to depict their vulnerability^17^.

Resilience was assessed with CD-RISC-25. This questionnaire depicts a measure of both psychological and physical resilience. Patients included in this cohort experienced both the physical stress of the COVID-19 disease as well as the psychological stress related to isolation, stigma, and not infrequently the experience of people who have died from the same disease. The cut off score of 75.5 validated in the general population had a ceiling effect in this cohort, therefore we chose an intra-cohort cut-off above the median (60.6) in order to be able to increase the sensitivity of the tool.

Both frailty and resilience dichotomous classification (supplementary table 1) were useful categories associated with PACS but some differences existed amongst the two. The former was specifically associated with sex, inflammatory biomarkers and geriatric syndromes, while resilience was associated with neurocognitive, psychological clusters and loneliness.

Apparently, the characterization of the four phenotypes was superior to identify the different prevalence of PACS with a clear increasing gradient from “fit and resilient” phenotype to the “frail and non-resilient” one. With this regard, the frail/resilient constructs, used in combination can measure patient’s vulnerability better than a single construct alone.

A gradient risk of frailty/resilience phenotypes was also observed in geriatric syndromes, Minor differences may be attributed to the relatively small group of the frail/resilience group. We may argue that the higher prevalence of geriatric syndromes across the 4 phenotypes may interfere with different aging trajectories in the 4 groups. Interestingly, non-resilience was associated with lower hand grip. This parameter is a well-known marker of sarcopenia, strongly associated with frailty. We speculate that sarcopenia may also be a trigger of lower resilience^31^. Further studies may address if therapeutic interventions on sarcopenia may improve both frailty and resilience.

Moreover, the 4 phenotypes better discriminated study outcomes. This is expected in consideration of the multi-dimensional constructs of QoL and IC, which capture simultaneously physical and psychological wellbeing. Radar graphs, visually depicted the gradient of severity of the 4 phenotypes and can be used in clinical setting to address individual patients scores compared to a reference population and to suggest potential areas of intervention. Prospective data may allow us to analyze the interaction between damage and repair mechanisms and explain why some individuals are aging “faster”. This may pave the way to further studies to explore pathways of accelerated aging in people hospitalized for severe COVID-19.

Logistic regression models for both EQ-5D-5L and SF-36, using fit/resilient as a reference, showed that frail/non resilient phenotype had the highest risk of lower HRQoL. In detail, when SF-36 was used as an outcome, frail/resilient phenotype had twofold higher odds of lower score than frail/resilient phenotype, indicating that frailty and resilience synergistically capture multiple domains of of HRQoL. On the contrary, when EQ-5D-5L was used as an outcome, two frail phenotypes had similar risk of lower HRQoL. Moreover, EQ-5D-5L was also able to depict that fit/non resilient phenotype had higher odds of total score < 89.7, suggesting that this measure may have optimal specificity to identify and distinguish clinical phenotypes of patients with PACS at greater risk of impaired QoL.

HR-QoL is rarely addressed in patients with PACS^32^ regardless the high burden of PACS clusters potentially interfering with well-being. Our results showed that PACS prevalence was 74.6%, which is in line with previous findings of 50–80% in hospitalized patients^4–8,33–36^. We described HRQoL both in continuous and categorical terms, by means of SF-36 questionnaires and EQ-5D-5L. An early UK study including 100 patients that were assessed 4–8 weeks after discharge, reported a clinically significant drop in EQ-5D-5L in 68.8% of participants in the intensive care units group and in 45.6% of participants in the ward group respectively^37^. One French study, comprising 120 patients, reported a decline in quality of life assessed with EQ-5D-5L in all domains, but without major differences between the patients previously hospitalized in wards and intensive care units^5^. Interestingly, similarly to our approach, both reports used radar representation of EQ-5D-5L. The additional value of our radar figures was the stratification of study outcomes according to diverse vulnerability groups by the frail/resilience phenotypes.

A Dutch study including 101 patients previously hospitalized with moderate and severe pneumonia, assessed the relationship between HR-QoL, measured with SF-36, and perceived dyspnea and pulmonary function. The findings suggest significant impairment across all SF-36 domains in comparison with general healthy population. However, the correlation of these domains with pulmonary function was weak, implying that HR-QoL is determined by more features than SF-36 may capture^38^. The additional value of our study is use of both EQ-5D-5L and SF-36 to determine HR-QoL. Our findings suggest that both scales were associated similarly with frailty-resilience phenotypes. Furthermore, HR-QoL was chosen as a relevant clinical outcome, as two definitions of PACS quote important impact of PACS symptoms on everyday functioning^2,39^.

This study also addressed intrinsic capacity that has been promoted by World Health Organization through the Integrated Care of Older People (ICOPE) strategy^27^. IC is usually described as “the composite of all physical and mental attributes on which an individual can draw”^19,40^. Both non-resilient phenotypes had a higher risk of impaired IC, underlying the importance of multidisciplinary and multidimensional evaluation of all five IC functional domains in people who are screened for PACS. In the current literature, IC construct is perceived as evolution of the frailty concept, that depicts the continuum of aging trajectory^19^. It is argued that IC trajectories in the COVID-19 era may be used as a tool to inform clinical decision making and proper interventions in the vulnerable populations^41^.

Several limitations can be acknowledged and are intrinsic to the observational and cross-sectional nature of the study design. Secondly, survival bias cannot be ignored, as some reports show that almost 30% of patients with COVID-19 who were discharged alive from the hospital, died shortly during the follow-up or were re-admitted to hospital for other reasons^42,43^. Thirdly, although our sample size is not small, the characteristics of our study population cannot be generalized to all people who had COVID-19 or are from different geographical areas. Fourthly, we included only people who were willing or able to complete electronic questionnaires, therefore bias related to digital divide cannot be excluded.

The point of strength of this study is the patient centered approach which attempted to characterized quality of life and well-being according to patients’ physical and psychological vulnerability.

In conclusion, these data shows that frailty-resilience construct characterizes health status and well-being of people previously hospitalized for severe COVID-19. Resilience is complementary to frailty in the identification of clinical phenotypes with different impacts on relevant clinical outcomes including different measures of wellness and HRQoL. Frailty and resilience may be evaluated in people with PACS to identify vulnerable individuals that require suitable interventions.

## Supplementary Information

Supplementary Information.

## Data Availability

The datasets used and/or analyzed during the current study available from the corresponding author on reasonable request.
