# Supplementary material for: Quality of life and intrinsic capacity in patients with post-acute COVID-19 syndrome is in relation to frailty and resilience phenotypes
Source: Sci Rep. 2023 Jun 2;13:8956. doi: 10.1038/s41598-023-29408-z (PMC10235830; doi:10.1038/s41598-023-29408-z)
Supplement: Supplementary file 1 — Supplementary Information. [file 41598_2023_29408_MOESM1_ESM.docx]

**Supplementary table 1***.* 37 items included in the Intrinsic capacity index.

| **PSYCHOSOCIAL** | | | |
| --- | --- | --- | --- |
| *1 = Employed*  *2 = Unemployed*  *3 = Retired* | **1** | What is your working status? | If employed, 1 point |
| **EMA mood**  *1 = Not at all*  *2 = Very slightly 3 = A little*  *4 = Quite a bit*  *5 = Extremely* | **2** | In this moment I feel: upset | If ≤ 3, 1 point |
|  | **3** | In this moment I feel: nervous | If ≤ 3, 1 point |
|  | **4** | In this moment I feel: enthusiastic | If ≥ 3, 1 point |
|  | **5** | In this moment I feel: active | If ≥ 3, 1 point |
| **EMA stress level**  *From 0 (as bad as it can be) to 10 (as good as it can be)* | **6** | How would you rate your current stress level? | If ≥ 3, 1 point |
| **EMA social** | **7** | Where are you now? | If “not at home", 1 point |
|  | **8** | Who are you with now? | If "not alone", 1 point |
|  | **9** | How many people have you socialized with so far today? | If “more than one”, 1 point |
| **VITALITY** | | | |
| **EMA eating**  *1 = Strongly disagree*  *2 = Disagree*  *3 = Neutral*  *4 = Agree*  *5 = Strongly agree* | **10** | Did you overeat during your last meal? | If ≤ 3, 1 point |
|  | **11** | Are you craving for a meal? | If ≤ 3, 1 point |
| *Yes or No* | **12** | Do you have a decrease in appetite and/or eat less than usual? | If no, 1 point |
| **Insomnia severity index** | | | |
| *0 = None*  *1 = Mild*  *2 = Moderate*  *3 = Severe*  *4 = Very severe* | **13** | Please, rate the current (i.e. last 2 weeks) severity of your insomnia problems regarding difficulty falling asleep. | If <15, 1 point |
|  | **14** | Please, rate the current (i.e. last 2 weeks) severity of your insomnia problems regarding difficulty staying asleep. |  |
|  | **15** | Please, rate the current (i.e. last 2 weeks) severity of your insomnia problems regarding problems waking up too early. |  |
| *0 = Very satisfied*  *1 = Satisfied*  *2 = Moderately satisfied*  *3 = Dissatisfied*  *4 = Very dissatisfied* | **16** | How satisfied/dissatisfied are you with your current sleep pattern? |  |
| *0 = Not at all*  *1 = A little*  *2 = Somewhat*  *3 = Much*  *4 = Very much* | **17** | How noticeable to others do you think your sleep problem is in terms of impairing the quality of your life? |  |
|  | **18** | How worried/distressed are you about your current sleep problem? |  |
|  | **19** | To what extent do you consider your sleep problem to interfere with your daily functioning (e.g. daytime fatigue, mood, ability to function at work/daily chores, concentration, memory, mood, etc). currently? |  |
| **EMA smoke**  *Yes or No* | **20** | Did you smoke in the last two hours? | If no, 1 point |
| **SHARE**  *1 = Very satisfied*  *2 = Somewhat satisfied*  *3 = Somewhat dissatisfied*  *4 = Very dissatisfied* | **21** | How satisfied are you with your life in general? | If ≤ 2, 1 point |
| **SHARE**  *1 = Never*  *2 = Rarely*  *3 = Sometimes*  *4 = Often* | **22** | How often do you have conflict with friends, co-workers, acquaintances? | If ≤ 2, 1 point |
| **LOCOMOTION** | | | |
| **EMA physical activity** | **23** | What were you doing right before filling this questionnaire? | If physical activity/exercise, 1 point |
| **IPAQ sitting** | **24** | During the last 7 days, how much time did you spend sitting during a day? | If less than 4 hours, 1 point |
| **IPAQ walking** | **25** | During the last 7 days, on how many days did you walk for at least 10 minutes at a time? | If ≥ 5 days, 1 point |
| **IPAQ moderate** | **26** | During the last 7 days, on how many days did you do moderate physical activities like gardening, cleaning, bicycling at a regular pace, swimming or other fitness activities? | If ≥ 2 days, 1 point |
| **IPAQ intense** | **27** | During the last 7 days, on how many days did you do vigorous physical activities like heavy lifting, heavier garden or construction work, chopping woods, aerobics, jogging/running or fast bicycling? | If ≥ 2 days, 1 point |
| **CONGNITION** | | | |
| **PAOFI Neurocognitive**  *1 = Almost always*  *2 = Very often*  *3 = Fairly often*  *4 = Once in a while*  *5 = Very infrequently*  *6 = Almost never* | **28** | Memory:  How often do you forget something that has been told to you within the last day or two? | If ≥ 3, 1 point |
|  | **29** | Language:  How often do you have difficulty thinking of the words (other than names) for what you want to say? | If ≥ 3, 1 point |
| **SENSORY** | | | |
| **Hearing**  *Yes or No* | **30** | Are you able to follow a one-on-one conversation (even with hearing aids, if used normally)? | If yes, 1 point |
|  | **31** | Are you able to follow a conversation in a group of 3 or more people (even with hearing aids, if used normally)? | If yes, 1 point |
| **Hearing**  *0 = Not at all*  *1 = A little*  *2 = Somewhat*  *3 = Much*  *4 = Very much* | **32** | If you have a hearing problem, how much does it interfere with your daily life? | If ≥ 2, 1 point |
| **Vision**  *Yes or No* | **33** | Are you able to recognize a face at a distance of about 4 meters (with glasses if used normally)? | If yes, 1 point |
|  | **34** | Can you read newspaper letters (with glasses if used normally)? | If yes, 1 point |
| **Vision**  *0 = Not at all*  *1 = A little*  *2 = Somewhat*  *3 = Much*  *4 = Very much* | **35** | If you have a vision problem, how much does this interfere with your daily life? | If ≥ 2, 1 point |
| **Taste**  *Yes or No* | **36** | Do you feel the flavours when you eat? | If yes, 1 point |
| **Smell**  *Yes or No* | **37** | Are you able to perceive smells? | If yes, 1 point |
|  |  |  |  |

| **Supplementary table 2.** Demographic, anthropometric and clinical characteristics and outcomes according to presence or absence of frailty. | | | |
| --- | --- | --- | --- |
|  | **Fit (FI <0.25)**  **N=160 (69%)** | **Frail (FI**  **≥0.25)**  **N=72 (31%)** | **p** |
| **Demographic, anthropometric and clinical characteristics at MPC visit** | | | |
| Age, years, median (Q1-Q3) [N_0_] | 59.0 (50.0 - 66.0) [160] | 58.0 (51.8 - 67.3) [72] | 0.20 |
| Male sex, N (%) | 105 (65.6%) | 36 (50.0%) | 0.03 |
| Body mass index, kg/m^2^, median (IQR) [N_0_] | 28.9 (25.8 - 31.7) [138] | 30.6 (26.4 - 34.7) [66] | 0.03 |
| ASCVD risk score, median (Q1-Q3) [N_0_] | 10.0 (4.9 - 20.3) [95] | 7.2 (2.6 - 11.6) [41] | 0.42 |
| **Physical activity, N (%)**  Low physical activity  Moderate physical activity  Intense physical activity | 89 (55.6%)  65 (40.6%)  6 (3.8%) | 67 (93.1%)  5 (6.9%)  0% | <0.001 |
| Metabolic syndrome, N (%) | 24 (15.0%) | 16 (22.2%) | 0.30 |
| Diabetes, N (%) | 17 (10.6%) | 9 (12.5%) | 0.92 |
| **PACS clusters** | | | |
| Respiratory cluster, N (%) | 71 (44.4%) | 57 (79.2%) | <0.001 |
| Neurocognitive cluster, N (%) | 43 (26.9%) | 39 (54.2%) | <0.001 |
| Musculoskeletal cluster, N (%) | 32 (20.0%) | 35 (48.6%) | <0.001 |
| Psychological cluster, N (%) | 41 (25.6%) | 38 (52.8%) | <0.001 |
| Sensory cluster, N (%) | 29 (18.1%) | 20 (27.8%) | 0.13 |
| Dermatologic cluster, N (%) | 23 (14.4%) | 19 (26.4%) | 0.04 |
| NAFLD cluster, N (%) | 60 (37.5%) | 33 (45.8%) | 0.60 |
| PACS diagnosis, N (%) | 107 (66.9%) | 66 (91.7%) | <0.001 |
| **Geriatric syndromes** | | | |
| Falls in the last year, N (%) | 14 (8.8%) | 21 (29.2%) | <0.001 |
| Polypharmacy, N (%) | 28 (17.5%) | 29 (40.3%) | <0.001 |
| Walked less in the last year, N (%) | 63 (39.4%) | 53 (73.6%) | <0.001 |
| Loneliness, N (%) | 19 (11.9%) | 25 (34.7%) | <0.001 |
| **OUTCOMES** | | | |
| **SF-36** |  |  |  |
| Overall SF-36 score, mean (±SD) [N_0_] | 58 (±20) [160] | 46 (±19) [72] | <0.001 |
| Health change, mean (±SD) [N_0_] | 38 (±23) [160] | 24 (±23) [72] | <0.001 |
| Energy/fatigue, median (Q1,Q3) [N_0_] | 60 (45 - 75) [160] | 40 (30 - 51) [72] | <0.001 |
| Well-being, mean (±SD) [N_0_] | 72 (60 - 81) [160] | 56 (44 - 68) [72] | <0.001 |
| Pain, mean (±SD) [N_0_] | 75 (±23) [160] | 53 (±27) [72] | <0.001 |
| Physical functioning, mean (±SD) [N_0_] | 81 (±18) [160] | 53 (±25) [72] | <0.001 |
| Role limitations due to physical health, mean (±SD) [N_0_] | 67 (±39) [160] | 32 (±39) [72] | <0.001 |
| Role limitations due to emotional problems, mean (±SD) [N_0_] | 72 (±35) [160] | 38 (±38) [72] | <0.001 |
| Social functioning, mean (±SD) [N_0_] | 73 (±23) [160] | 55 (±23) [72] | <0.001 |
| **EQ-5D-5L** |  |  |  |
| EQ-5D-5L, self-reported health score, mean (±SD) | 70 (±15) [160] | 60 (±14) [72] | <0.001 |
| EQ-5D-5L score, mean (±SD) | 86 (±12) [160] | 65 (±25) [72] | <0.001 |
| **Intrinsic capacity** |  |  |  |
| Cognition, mean (±SD) [N_0_] | 89 (±27) [160] | 79 (±33) [72] | 0.02 |
| Mobility, mean (±SD) [N_0_] | 43 (±28) [160] | 28 (±26) [72] | <0.001 |
| Psychosocial, mean (±SD) [N_0_] | 72 (±16) [160] | 63 (±15) [72] | <0.001 |
| Sensory, mean (±SD) [N_0_] | 95 (±9) [160] | 92 (±11) [72] | 0.02 |
| Vitality, mean (±SD) [N_0_] | 85 (±16) [160] | 82 (±16) [72] | 0.15 |
|  | | | |

| **Supplementary table 3.** Demographic, anthropometric and clinical characteristics and outcomes according to resilience. | | | |
| --- | --- | --- | --- |
|  | **Resilient**  **(CD-RISC-25 ≥60)**  **N=118 (50.9%)** | **Non-resilient**  **(CD-RISC-25 <60)**  **N=114 (49.1%)** | **p** |
| **Demographic, anthropometric and clinical characteristics at MPC visit** | | | |
| Age, years, median (Q1-Q3) [N_0_] | 59.5 (51.3 - 67.0) [118] | 58.0 (50.0 - 66.0) [114] | 0.44 |
| Male sex, N (%) | 77 (65.3%) | 64 (56.1%) | 0.20 |
| Body mass index, kg/m^2^, median (IQR) [N_0_] | 29.3 (26.0 - 32.2) [102] | 29.3 (25.6 - 32.6) [102] | 0.88 |
| ASCVD risk score, median (Q1-Q3) [N_0_] | 9.6 (5.0 - 17.6) [69] | 8.1 (3.8 - 14.1) [67] | 0.87 |
| **Physical activity, N (%)**  Low physical activity  Moderate physical activity  Intense physical activity | 73 (61.9%)  41 (34.8%)  4 (3.4%) | 83 (72.8%)  29 (25.4%)  2 (1.8%) | 0.19 |
| Metabolic syndrome, N (%) | 17 (14.4%) | 23 (20.2%) | 0.44 |
| Diabetes, N (%) | 14 (11.9%) | 12 (10.5%) | 0.88 |
| **PACS clusters** | | | |
| Respiratory cluster, N (%) | 54 (45.8%) | 74 (64.9%) | 0.005 |
| Neurocognitive cluster, N (%) | 31 (26.3%) | 51 (44.7%) | 0.005 |
| Musculoskeletal cluster, N (%) | 30 (25.4%) | 37 (32.5%) | 0.30 |
| Psychological cluster, N (%) | 33 (28.0%) | 46 (40.4%) | 0.06 |
| Sensory cluster, N (%) | 20 (17.0%) | 29 (25.4%) | 0.15 |
| Dermatologic cluster, N (%) | 16 (13.6%) | 26 (22.8%) | 0.10 |
| NAFLD cluster, N (%) | 43 (36.4%) | 50 (43.9%) | 0.34 |
| PACS diagnosis, N (%) | 81 (68.6%) | 92 (80.7%) | 0.05 |
| **Geriatric syndromes** | | | |
| Falls in the last year, N (%) | 19 (16.1%) | 16 (14.0%) | 0.80 |
| Polypharmacy, N (%) | 24 (20.3%) | 33 (29.0%) | 0.17 |
| Walked less in the last year, N (%) | 54 (45.8%) | 62 (54.4%) | 0.24 |
| Loneliness, N (%) | 14 (11.9%) | 30 (26.3%) | 0.008 |
| **Outcomes**  **SF-36** | | | |
| Overall SF-36 score, mean (±SD) [N_0_] | 62 (±19) [118] | 47 (±19) [114] | <0.001 |
| Health change, mean (±SD) [N_0_] | 39 (±25) [118] | 29 (±22) [114] | 0.001 |
| Energy/fatigue, median (Q1,Q3) [N_0_] | 60 (45 - 75) [118] | 50 (35 - 60) [114] | <0.001 |
| General health, mean (±SD) [N_0_] | 72 (61 - 84) [118] | 56 (48 - 72) [114] | <0.001 |
| Pain, mean (±SD) [N_0_] | 74 (±24) [118] | 62 (±28) [114] | <0.001 |
| Physical functioning, mean (±SD) [N_0_] | 76 (±24) [118] | 68 (±24) [114] | 0.02 |
| Role limitations due to physical health, mean (±SD) [N_0_] | 61 (±41) [118] | 51 (±42) [114] | 0.06 |
| Role limitations due to emotional problems, mean (±SD) [N_0_] | 68 (±37) [118] | 54 (±41) [114] | 0.008 |
| Social functioning, mean (±SD) [N_0_] | 74 (±25) [118] | 62 (±22) [114] | <0.001 |
| **EQ-5D-5L** | | | |
| EQ-5D-5L, self-reported health score, mean (±SD) | 71 (±15) [118] | 62 (±15) [114] | <0.001 |
| EQ-5D-5L score, mean (±SD) | 85 (±15) [118] | 74 (±22) [114] | <0.001 |
| **Intrinsic capacity** | | | |
| Cognition, mean (±SD) [N_0_] | 89 (±28) [118] | 82 (±31) [114] | 0.07 |
| Mobility, mean (±SD) [N_0_] | 43 (±28) [118] | 33 (±28) [114] | 0.01 |
| Psychosocial, mean (±SD) [N_0_] | 76 (±14) [118] | 63 (±16) [114] | <0.001 |
| Sensory, mean (±SD) [N_0_] | 95 (±10) [118] | 94 (±10) [114] | 0.58 |
| Vitality, mean (±SD) [N_0_] | 88 (±14) [118] | 80 (±17) [114] | <0.001 |
|  | | | |
